# Supplementary material for: Temporal processing and context dependency in Caenorhabditis elegans response to mechanosensation
Source: eLife. 2018 Jun 26;7:e36419. doi: 10.7554/eLife.36419 (PMC6054533; doi:10.7554/eLife.36419)
Supplement: Transparent reporting form [file elife-36419-transrepform.docx]

***eLife’s* transparent reporting form**

We encourage authors to provide detailed information *within their submission* to facilitate the interpretation and replication of experiments. Authors can upload supporting documentation to indicate the use of appropriate reporting guidelines for health-related research (see [EQUATOR Network](http://www.equator-network.org/%20)), life science research (see the [BioSharing Information Resource](https://biosharing.org/)), or the [ARRIVE guidelines](http://www.plosbiology.org/article/info:doi/10.1371/journal.pbio.1000412) for reporting work involving animal research. Where applicable, authors should refer to any relevant reporting standards documents in this form.

If you have any questions, please consult our Journal Policies and/or contact us: [editorial@elifesciences.org](mailto:editorial@elifesciences.org).

**Sample-size estimation**

- You should state whether an appropriate sample size was computed when the study was being designed
- You should state the statistical method of sample size computation and any required assumptions
- If no explicit power analysis was used, you should describe how you decided what sample (replicate) size (number) to use

No explicit power analysis was performed prior to the experiment partly because, to our knowledge, there is not yet a well-established method for *a priori* estimating sample size for the reverse correlation experiments we performed. Instead we recorded three orders of magnitude more animal-hours of behavior during stimulation than previous *C. elegans* optogenetic studies. We were satisfied with the sample size because it yielded many kernels that passed our shuffled significance test (see subsubsection “Kernel Significance” in the “Reverse Correlation” subsection in the “Methods” section).

Please outline where this information can be found within the submission (e.g., sections or figure legends), or explain why this information doesn’t apply to your submission:

**Replicates**

- You should report how often each experiment was performed
- You should include a definition of biological versus technical replication
- The data obtained should be provided and sufficient information should be provided to indicate the number of independent biological and/or technical replicates
- If you encountered any outliers, you should describe how these were handled
- Criteria for exclusion/inclusion of data should be clearly stated
- High-throughput sequence data should be uploaded before submission, with a private link for reviewers provided (these are available from both GEO and ArrayExpress)

Please outline where this information can be found within the submission (e.g., sections or figure legends), or explain why this information doesn’t apply to your submission:

For each experimental series, Table 2 lists the number of plates recorded, the average number of animals per plate, the number of days on which recordings were performed, the total number of animal-hours recorded, and the total number of animal-stimulus presentations. Each figure caption similarly notes the number of animal-stimulus presentations per condition.

**Statistical reporting**

- Statistical analysis methods should be described and justified
- Raw data should be presented in figures whenever informative to do so (typically when N per group is less than 10)
- For each experiment, you should identify the statistical tests used, exact values of N, definitions of center, methods of multiple test correction, and dispersion and precision measures (e.g., mean, median, SD, SEM, confidence intervals; and, for the major substantive results, a measure of effect size (e.g., Pearson's r, Cohen's d)
- Report exact p-values wherever possible alongside the summary statistics and 95% confidence intervals. These should be reported for all key questions and not only when the p-value is less than 0.05.

Please outline where this information can be found within the submission (e.g., sections or figure legends), or explain why this information doesn’t apply to your submission:

Statistical analysis is described in the methods, specifically in the sections labeled “Reverse correlation” and “Calculating transition rates.” Number of stimulus presentations, or number of observed transitions are listed in each figure or in the figure caption. P-values are reported in figure captions, or in dedicated tables, as in Figure 8 - Figure supplements 2 and 4.

(For large datasets, or papers with a very large number of statistical tests, you may upload a single table file with tests, Ns, etc., with reference to sections in the manuscript.)

**Group allocation**

- Indicate how samples were allocated into experimental groups (in the case of clinical studies, please specify allocation to treatment method); if randomization was used, please also state if restricted randomization was applied
- Indicate if masking was used during group allocation, data collection and/or data analysis

Please outline where this information can be found within the submission (e.g., sections or figure legends), or explain why this information doesn’t apply to your submission:

Group allocation does not apply to this investigation.

**Additional data files (“source data”)**

- We encourage you to upload relevant additional data files, such as numerical data that are represented as a graph in a figure, or as a summary table
- Where provided, these should be in the most useful format, and they can be uploaded as “Source data” files linked to a main figure or table
- Include model definition files including the full list of parameters used
- Include code used for data analysis (e.g., R, MatLab)
- Avoid stating that data files are “available upon request”

Please indicate the figures or tables for which source data files have been provided:

All analysis code is available on GitHub and the URL is listed in the main text. MATLAB files containing behavior timeseries data for the stimulus and the behavior of every animal at every time point in every experiment has been posted on FigShare and a DOI is provided in the text. All raw imaging data (>1 TB) is in the process of being uploaded to IEEE DataPorts where it will be freely available to all. A DOI to this repository has been provided in the text.
